# Increased liver stiffness promotes hepatitis B progression by impairing innate immunity in CCl4-induced fibrotic HBV^+^ transgenic mice

**DOI:** 10.3389/fimmu.2023.1166171

**Published:** 2023-08-03

**Authors:** Grace Bybee, Youra Moeun, Weimin Wang, Kusum K. Kharbanda, Larisa Y. Poluektova, Srivatsan Kidambi, Natalia A. Osna, Murali Ganesan

**Affiliations:** ^1^ Research Service, Veterans Affairs Nebraska-Western Iowa Health Care System, Omaha, NE, United States; ^2^ Department of Internal Medicine, University of Nebraska Medical Center, Omaha, NE, United States; ^3^ Department of Chemical and Biomolecular Engineering, University of Nebraska at Lincoln, Lincoln, NE, United States; ^4^ Department of Pharmacology and Experimental Neuroscience, University of Nebraska Medical Center, Omaha, NE, United States

**Keywords:** HBV, liver stiffness, innate immunity, alcohol, osteopontin (OPN)

## Abstract

**Background:**

Hepatitis B virus (HBV) infection develops as an acute or chronic liver disease, which progresses from steatosis, hepatitis, and fibrosis to end-stage liver diseases such as cirrhosis and hepatocellular carcinoma (HCC). An increased stromal stiffness accompanies fibrosis in chronic liver diseases and is considered a strong predictor for disease progression. The goal of this study was to establish the mechanisms by which enhanced liver stiffness regulates HBV infectivity in the fibrotic liver tissue.

**Methods:**

For *in vitro* studies, HBV-transfected HepG2.2.15 cells were cultured on polydimethylsiloxane gels coated by polyelectrolyte multilayer films of 2 kPa (soft) or 24 kPa (stiff) rigidity mimicking the stiffness of the healthy or fibrotic liver. For *in vivo* studies, hepatic fibrosis was induced in C57Bl/6 parental and HBV+ transgenic (HBVTg) mice by injecting CCl4 twice a week for 6 weeks.

**Results:**

We found higher levels of HBV markers in stiff gel-attached hepatocytes accompanied by up-regulated OPN content in cell supernatants as well as suppression of anti-viral interferon-stimulated genes (ISGs). This indicates that pre-requisite “fibrotic” stiffness increases osteopontin (OPN) content and releases and suppresses anti-viral innate immunity, causing a subsequent rise in HBV markers expression in hepatocytes. *In vitro* results were corroborated by data from HBVTg mice administered CCl4 (HBVTg CCl4). These mice showed higher HBV RNA, DNA, HBV core antigen (HBcAg), and HBV surface antigen (HBsAg) levels after liver fibrosis induction as judged by a rise in Col1a1, SMA, MMPs, and TIMPs mRNAs and by increased liver stiffness. Importantly, CCl4-induced the pro-fibrotic activation of liver cells, and liver stiffness was higher in HBVTg mice compared with control mice. Elevation of HBV markers and OPN levels corresponded to decreased ISG activation in HBVTg CCl4 mice vs HBVTg control mice.

**Conclusion:**

Based on our data, we conclude that liver stiffness enhances OPN levels to limit anti-viral ISG activation in hepatocytes and promote an increase in HBV infectivity, thereby contributing to end-stage liver disease progression.

## Introduction

In approximately 10% of cases, Hepatitis B virus (HBV) infection becomes chronic and progresses from steatosis, hepatitis, and fibrosis to end-stage liver diseases such as cirrhosis and hepatocellular carcinoma (HCC). Approximately 400 million individuals worldwide have chronic hepatitis B (CHB) and almost one million die annually from complications due to persistent infection, liver cirrhosis, and HCC. (1–5) Despite the availability of an efficient vaccine, chronic HBV infection persists. (6) In adults, horizontal transmission mainly results in self-limiting acute HBV infection, while vertical transmission leads to CHB. (7) Current treatments include interferon-based therapies and several nucleos(t)ide analogs. While nucleos(t)ide analogs rarely cure CHB, they suppress viral replication which slows down disease progression that would otherwise eventually manifest as end-stage liver disease. (8) HBV is still the most frequent cause of liver fibrosis (9), so current treatment strategies are focused on the long-term suppression of HBV replication. Hepatic stellate cells (HSCs) activation is a crucial pathogenic feature of liver fibrosis that makes it a hotspot in this research field. HBV triggers HSCs activation via DAMPs release and the host antiviral immune response, leading to chronic inflammation. (10) However, unlike the hepatitis C virus (HCV), the mechanisms of liver fibrosis development remain less defined in CHB.

HBV is not cytotoxic, and the infected hepatocytes are eliminated by immune mechanisms. The first line of anti-viral defense, which secures the effective adaptive immune response, is related to innate immunity, specifically to the activation of anti-viral interferon-stimulated genes (ISGs) to resist/control HBV infection in hepatocytes, thereby preventing its spread and persistence. HBV infects only hepatocytes, which are removed by the immune system, causing continuous cycles of hepatocyte damage followed by tissue repair. This requires the deposition of the extracellular matrix with subsequent progressive liver fibrosis over time. (11, 12) An increased stromal stiffness accompanies fibrosis in chronic liver diseases (13) and serves as a strong predictor of disease progression to end-stage liver diseases (14), including hepatitis B. (15) Clinically viewed as the indicator of organ fibrosis, tissue stiffness increases remarkably in line with deposition, cross-linking, and reconstruction of the matrix. (16) Recent studies have shown that matrix stiffness plays a pivotal role in fibroblast activation and outcomes of pathological fibrosis. (16, 17)

It has been suggested that matrix stiffness plays a role in the regulation of some biochemical markers expression, such as osteopontin (OPN). (18, 19) OPN is a matricellular glycophosphoprotein produced by different cells and is associated with the synthesis and accumulation of the extracellular matrix (ECM) and, therefore, promotes fibrosis progression. (20) Importantly, serum osteopontin (OPN) concentrations were found to be significantly increased in HBV-infected patients and patients with hepatocellular carcinoma (HCC). (21) In addition, the role of OPN has been elucidated in inflammatory liver disorders, such as T cell−mediated hepatitis and alcoholic and nonalcoholic disorders. (22–24) However, to date, there are no thorough investigations on liver stiffness, the role of OPN expression, and HBV infection pathogenesis. The aim of this study was to establish the mechanisms by which enhanced liver stiffness regulates HBV infection levels in liver fibrosis.

In this study, we aimed to analyze both *in vitro* and *in vivo* evidence of a liver stiffness-mediated increase of HBV markers in HBV-transfected HepG2.2.15 cells and in HBV-infected fibrotic liver. We hypothesize that a pre-requisite “fibrotic” stiffness increases OPN levels in hepatocytes and suppresses anti-viral innate immunity, causing a subsequent rise in the expression of HBV markers. For *in vitro* studies, HBV-transfected HepG2.2.15 cells were cultured on an innovative biomimetic platform named BEASTS (Bio-Engineered Adhesive Siloxane substrate with Tunable Stiffness) that utilizes a polydimethylsiloxane (PDMS) substrate, along with polyelectrolyte multilayer film-coating technology. By employing this approach, we can engineer substrates that closely mimic physiological (2 kPa) and fibrotic (24 kPa) stiffnesses of the liver (25–29). *In vitro* results were confirmed by *in vivo* studies, in which hepatic fibrosis was induced in C57Bl/6 parental and HBV^+^ transgenic (HBVTg) mice by injecting CCl4 twice a week for 6 weeks to mimic fibrotic liver. This current study will help in identifying the mechanisms by which liver tissue stiffness regulates the pathogenesis of HBV infection allowing for the detection of new targets for treatment.

## Materials and methods

### Reagents and media

High glucose Dulbecco’s Modified Eagle Medium (DMEM) and fetal bovine serum were purchased from Invitrogen (Carlsbad, CA). Trizol was acquired from Life Technologies (Carlsbad, CA). All RNA isolation, cDNA synthesis, and RT-PCR reagents and primers (human and mouse) were obtained from ThermoFisher Scientific (Carlsbad and Foster City, CA). The DNA isolation kit was obtained from Qiagen (Louisville, KY). Human Osteopontin DuoSet and Mouse Osteopontin DuoSet ELISA kits were obtained from R&D Systems (Minneapolis, MN). Interferon α (IFNα) was purchased from Merck Sharp & Dohme Corp (NJ, USA; cat# NDC 0085-0571-02). We used the following antibodies: anti-Hepatitis B core antigen and anti-Hepatitis B surface antigen from INNOVEX Biosciences Inc (Richmond, CA), αSMA from Abcam (Cambridge, MA), anti-pSTAT-1, cleaved caspase 3 and anti-USP18 from Cell Signaling Technology (Beverly, MA), and anti-STAT-1 and Anti-β actin from Santa Cruz Biotechnology Inc. (Santa Cruz, CA).

### Cell culture and treatments

In this study, we used HepG2.2.15 cells that were stably transfected with HBV and were able to replicate the virus and produce viral particles (HBV genotype D). (30) HepG2.2.15 cells were cultured on an innovative biomimetic platform named BEASTS (Bio-Engineered Adhesive Siloxane substrate with Tunable Stiffness) based on polydimethylsiloxane (PDMS) substrate in combination with polyelectrolyte multilayer film-coating technology to engineer substrates mimicking physiologic (2 kPa) and fibrotic liver stiffness (24 kPa). (25–29) In summary, Sylgard 527 and Sylgard 184 were mixed in specific weight ratios, as outlined in **Table 1**, and crosslinked according to the manufacturer’s instructions. For Sylgard 527, components A and B were mixed in equal parts, while Sylgard 184 was mixed in a ratio of 10:1 of elastomer to the cross-linking agent. The two mixtures were then combined in a determined weight ratio and poured into 12-well tissue culture plates to crosslink at 65 °C. The resulting PDMS gels were then rendered hydrophilic through a 7-minute oxygen plasma treatment before being coated with PEMs using poly(diallyldimethylammoniumchloride) (PDAC) (Mw∼100,000-200,000) as a 20 wt % solution, and sulfonated poly(styrene) and sodium salt (SPS) (Mw∼70,000) polymer solutions. This was achieved by immersing the PDMS substrates in alternating polycation and polyanion solutions to form multiple layers and was repeated five times. The resulting platform was then UV-sterilized overnight before being used to study the effects of stiffness on HBV-infected hepatocytes in a non-invasive environment. To study the type-1 IFNα signaling, cells were treated or not with interferon-alpha (IFNα) for 8hrs at 400 units concentration.

**Table 1:** Young’s Modulus of PEM-coated PDMS substrates used for primary hepatocyte culture was determined using the indentation load technique.

Substrate	Young’s Modulus (in KPa)
Soft (100% Sylgard 527)	2.4 ± 0.03
Stiff (91% wt Sylgard 527 + 9% wt Sylgard 184)	24.2 ± 0.03

### Recombinant osteopontin treatment

To mimic the overexpression of osteopontin, we used recombinant human osteopontin (rOPN) Protein from R & D Systems, Catalog #: 1433-OP. We pre-treated the cells in the presence or absence of rOPN at 100 nM for 24hrs, and IFNα was applied for the last 8 hours (400 units) to induce the IFNα signaling.

### Osteopontin siRNA transfection

Transfection was done using the protocol from the manufacturer (OriGene, Rockville, MD) with control (scrambled) or osteopontin siRNA (Cat#SR321880 SPP1). The efficacy of transfection was monitored for 48hrs for the osteopontin mRNA expression by RT-PCR. Human OPN Primer (Hs00959010_m1) was gotten from Thermo Fisher, Catalog #: 4331182.

### RNA and DNA isolation, real-time PCR, and ddPCR

Total RNA was isolated from cells using Trizol Reagent. DNA was isolated from cells using DNeasy Blood and Tissue kit (Qiagen, Louisville, KY) following the manufacturer’s instructions. RT-PCR was used to measure HBV RNA, OAS1, ISG15, APOBEC3G, USP18, Col1A1, TGFβ1, α-SMA, MMP2, and MMP9 mRNA levels in cells as described previously (31). ddPCR was used to quantify HBV DNA levels in HepG2.2.15 cells as previously described (32) using the following primers and probes: HBV sense (5′- CGA CGT GCA GAG GTG AAG-3′), antisense (5′- CAC CTC TCT TTA CGC GGA CT-3′) primers, and HBV probe (5′-/56-FAM/ATC TGC CGG/ZEN/ACC GTG TGC AC/3IABkFQ/-3′).

### ELISA

HBV surface antigen (HBsAg) and human osteopontin were measured in cell media by ELISA using LSBio Kit (LifeSpan Biosciences, Inc, Seattle, WA) and DuoSet Kit (R&D Systems, Minneapolis, MN), respectively.

### Immunoblotting (western blot)

Cell lysates prepared in 0.5 M EDTA, 2 M Tris, 20 mM Na_3_VO_4_, 200 mM Na_4_P_2_O_7_, 100 mM PMSF, 1 M NaF, 20% Triton X-100, and aprotinin, pH 7 were separated and subjected to the immunoblotting technique as previously described (32, 33). Blots were developed using the Odyssey infrared imaging system, and the protein bands were quantified using the Li-Cor software (Li-Cor Bioscience, Lincoln, NE).

### *In vivo* studies

#### Experimental manipulations on HBV+ transgenic mice

HBV+ transgenic (HBVTg) mice are a specially designed strain that carry the 1.3mer, over-length wild-type HBV genome, which expresses all viral proteins except the X protein. These mice were obtained from Dr. Jing-Hsiung James Ou, Professor of Molecular Microbiology and Immunology, University of Southern California, Los Angeles (MTA on 04/21/2021). The DNA fragment used for producing the transgenic mice starts from nucleotide (nt) 1043, which is located upstream of the ENI enhancer and the X promoter, and terminates at nt 1987 located downstream of the unique poly(A) site. Sixteen mice were split equally into four groups (four mice per group): control C57Bl/6 (HBV-), C57Bl/6 with induced hepatic fibrosis (CCl4), control HBVTg (HBV+), and HBVTg with induced hepatic fibrosis (HBV+CCl4). All animals received humane care, and all methods were carried out in accordance with the Guide for the Care and Use of Laboratory Animals and were approved by the Institutional Animal Care and Use Committee. Hepatic fibrosis was induced in male and female C57BL/6J and HBVTg (HBV+) mice using CCl4 as described (34, 35). Briefly, CCl4 was diluted 1:7 in sunflower oil and injected intraperitoneally (i.p.) twice per week at a dose of 1 μl/g body weight (0.125 μl/g CCl4) for 6 weeks (35).

#### Serum enzyme measurements

The clinical laboratory at the VA NWIHCS analyzed the activity of alanine aminotransferase (ALT) and aspartate aminotransferase (AST).

#### RNA and DNA isolation, Real-time PCR, and ddPCR

Total RNA was isolated from mouse liver tissue using Trizol Reagent; DNA was isolated using DNeasy Blood and Tissue kit (Qiagen, Louisville, KY) following the manufacturer’s instructions. RT-PCR was used to measure HBV RNA, Col1a1, α-SMA, TIMP1, TIMP2, MMP2, MMP9, MMP13, and OPN mRNA levels in liver tissue as described previously (33). ddPCR was applied to quantify HBV DNA levels as previously described (33) using the following primers and probes: HBV sense (5′- CGA CGT GCA GAG GTG AAG-3′), antisense (5′- CAC CTC TCT TTA CGC GGA CT-3′) primers, and HBV probe (5′-/56-FAM/ATC TGC CGG/ZEN/ACC GTG TGC AC/3IABkFQ/-3′).

#### Histology and immunohistochemistry

To visualize collagen accumulation, sections of paraffin-embedded liver tissue were subjected to picrosirius red staining (36). For the immunohistochemical detection of α-smooth muscle actin (α-SMA), HBcAg, and HBsAg, sections were heated in antigen unmasking solution (Vector Labs, Burlingame, CA, USA), then probed with respective antibodies, and visualized using a Keyence BZ-X810 fluorescence microscope. Staining intensity in the captured images was quantified using the Keyence BZ-X810 Analyzer software. At least, 10 non-overlapping fields per low magnification (20X) section were analyzed.

#### ELISA

Mouse osteopontin DuoSet Kit (R&D Systems, Minneapolis, MN) was used to measure osteopontin levels in the media and serum.

#### Mechanical analysis of liver tissue

To determine the compressive Young’s modulus of the liver tissue, a microscale mechanical testing apparatus called the CellScale BioTester (Waterloo, Canada) was used. The samples were equilibrated at 37 °C and preconditioned for two cycles at 0.8 μm/s strain rate to eliminate hysteresis effects before each measurement. Compression was then performed at 10% strain with 30 s compression followed by a 2-second hold and 10-second recovery. The force versus displacement curve was analyzed using a custom MATLAB code to calculate Young’s modulus by measuring the slope of the curve.

### Statistical analyses

The data were expressed as mean values ± standard error. A one-way analysis of variance (ANOVA) was performed on the multiple groups of this study and a comparison was made using a Tukey *post-hoc* test. A Student’s t-test was used to compare two groups at a time. Results were considered significant at a probability value of 0.05 or less.

## Results

### Increased matrix stiffness upregulates HBV infection markers and OPN levels in HBV-transfected HepG2.2.15 cells

**Figure 1:**
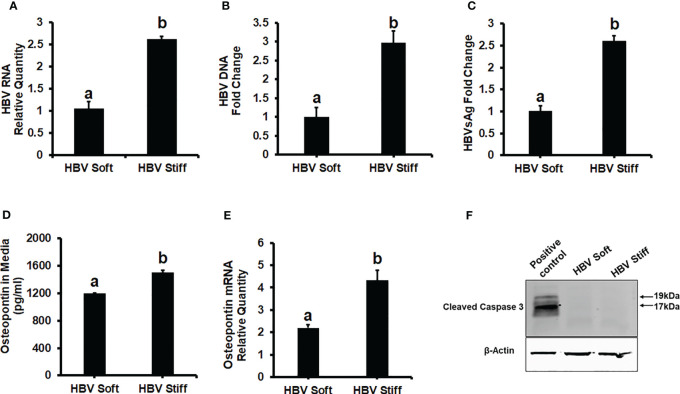
Increased matrix stiffness upregulates HBV infection markers and osteopontin levels in HBV-transfected HepG2.2.15 cells: Cells were plated in both soft and stiff plates for 48hrs. **(A)** HBV RNA levels were measured by Real-Time PCR. **(B)** HBV DNA levels were measured by Droplet Digital™ PCR (ddPCR). GAPDH was used as an internal control for HBV RNA and HBV DNA. **(C)** A Sandwich Elisa kit was used to measure HBsAg levels. **(D, E)** Osteopontin protein and mRNA levels were measured by Elisa and Real-Time PCR. **(F)** Cleaved caspase 3 protein expression was detected by immunoblotting in cell lysates for apoptosis. Equal (20 ug) amounts of protein were loaded in each lane. β-Actin was used as an internal control. Acetaldehyde-generating system (AGS)-treated HepG2 cell lysates were used as the positive control. Data are from three independent experiments presented as Mean ± SEM. Bars marked with the same letter are not significantly different from each other; bars with different letters are significantly different (p ≤0.05).

To investigate the role of increased matrix stiffness in the regulation of HBV expression in HepG2.2.15 cells, we quantified HBV RNA and DNA by RT-PCR and ddPCR, respectively, and used ELISA to quantify HBsAg levels (**Figures 1A-C**). We found that plating the cells at the increased matrix stiffness upregulated HBV markers by approximately 2.5-fold as evident from the results of stiff gel- (25 kPa) vs soft gel-attached cells. Enhanced matrix stiffness also increased OPN expression in these cells, and there is a correlation between osteopontin (OPN) levels and the expression of HBV markers. We measured OPN protein levels in the cell media and mRNA expression in cells and observed that higher levels of HBV markers in fibrotic stiff gel-attached hepatocytes were accompanied by up-regulated OPN protein content (p<0.05) in cell supernatants as well as OPN mRNA expression in cells (**Figures 1D, E**). In addition, we confirmed that there was no apoptotic cell death in fibrotic stiffness gel-attached HepG2.2.15 cells (**Figure 1F**).

### Increased matrix stiffness suppresses IFNα signaling in HBV-transfected HepG2.2.15 cells

**Figure 2:**
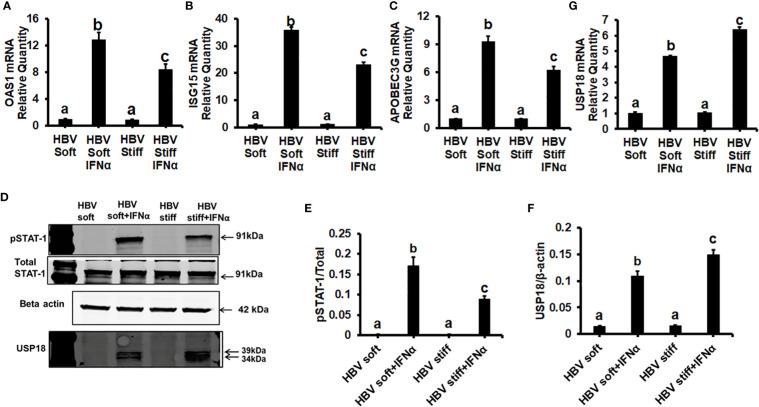
Increased matrix stiffness suppresses IFNα signaling in HBV transfected HepG2.2.15 cells: HepG2.2.15 cells were either treated or not treated with IFNα for the last 8 hours (ISGs), 1 hr (pSTAT-1), and 24 hours (USP18 protein). RT-PCR analysis was done for mRNA expression of IFN-induced genes. **(A)** 2′–5′ oligoadenylate synthetase 1 (OAS1); **(B)** Interferon-stimulated gene 15 (ISG15); **(C)** Apolipoprotein B Editing Complex (APOBEC3); **(G)** USP18. GAPDH acted as an internal control. **(D)** STAT-1 phosphorylation (pSTAT-1) and USP18 protein were measured by immunoblotting. Total STAT-1 was used to normalize the data and β-actin was also used as an internal control. **(E, F)** Quantification of immunoblotting data. Data are from three independent experiments presented as Mean ± SEM. Bars marked with the same letter are not significantly different from each other; bars with different letters are significantly different (P ≤0.05).

Next, we tested whether the up-regulation of HBV infection markers was related to suppressed anti-viral interferon-stimulated genes and the impairment of IFNα-signaling via the JAK-STAT1 pathway. In this regard, in healthy (2 kPa) and fibrotic stiffness (25 kPa) gel-attached HepG2.2.15 cells, we measured mRNA expression of ISGs *(OAS1*, ISG15, and *APOBEC3G)* as well as STAT1 phosphorylation (Immunoblotting) in response to IFNα. We found that fibrotic stiffness gel-attached cells show a significant decrease (p<0.05) in ISGs mRNA expression (**Figures 2A–C**) and also a 2-fold reduction in the pSTAT1/STAT1 ratio (**Figures 2D, E**), when compared to the healthy stiffness gel-attached HBV-expressing cells, suggesting that fibrotic stiffness suppresses downstream events of IFNα signaling. In addition to this, we also found that both mRNA (**Figure 2G**) and protein expression (**Figures 2D, F**) of USP18, a negative regulator of IFN signaling, were significantly increased in fibrotic stiffness gel-attached HepG2.2.15 cells when compared to healthy stiffness gel-attached cells.

### OPN increases the expression of HBV markers by suppressing IFNα-inducible anti-viral genes activation in soft gel-attached HBV+ transfected HepG2.2.15 cells

**Figure 3:**
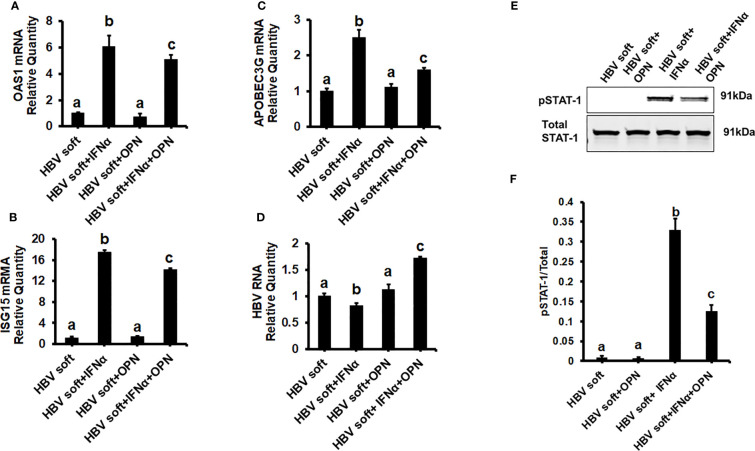
Recombinant OPN treatment increases the expression of HBV marker by suppressing IFNα-inducible anti-viral genes activation in soft gel (2 KPa) attached HBV+ transfected HepG2.2.15 cells: To mimic overexpression, we treated soft (2 kPa) gel-attached HBV transfected HepG2.2.15 cells with rOPN for 24hrs. RT-PCR analysis was done for mRNA expression of IFN-induced genes and HBV RNA. **(A)** 2′–5′ oligoadenylate synthetase 1 (OAS1); **(B)** Interferon-stimulated gene 15 (ISG15); **(C)** Apolipoprotein B Editing Complex (APOBEC3); **(D)** HBV RNA. GAPDH acted as an internal control. **(E)** STAT-1 phosphorylation (pSTAT-1) was measured by immunoblotting. Total STAT-1 was used to normalize the data. **(F)** Quantification of immunoblotting data. Data are from three independent experiments presented as Mean ± SEM. Bars marked with the same letter are not significantly different from each other; bars with different letters are significantly different (P ≤0.05).

**Figure 4:**
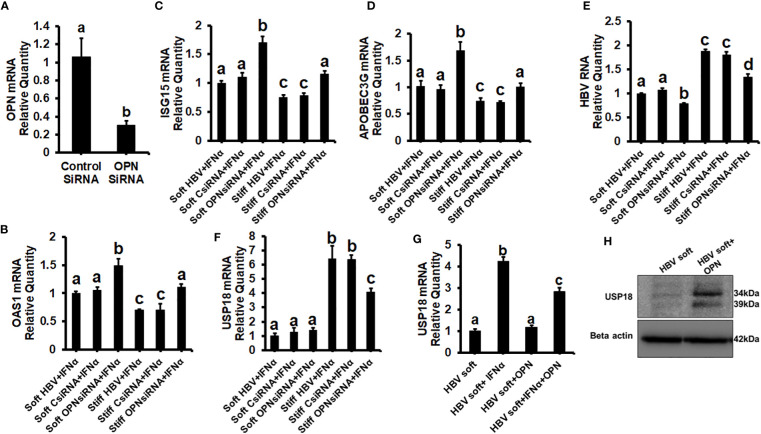
Silencing of OPN decreases expression of HBV marker by increasing IFNα-inducible anti-viral genes activation in soft gel (2 KPa)-attached HBV+ transfected HepG2.2.15 cells, and USP18 is partially responsible for the regulation of HBV infection markers by OPN in fibrotic stiffness gel-attached HBV+ transfected HepG2.2.15 cells: We silenced OPN by specific siRNA transfection, in soft (2 kPa) and stiff (25 kPa) gel-attached HBV transfected HepG2.2.15 cells. RT-PCR analysis was done for mRNA expression of IFN-induced genes and HBV RNA. **(A)** Transfection efficiency of OPN silencing and OPN mRNA; **(B)** 2′–5′ oligoadenylate synthetase 1 (OAS1); **(C)** Interferon-stimulated gene 15 (ISG15); **(D)** Apolipoprotein B Editing Complex (APOBEC3); **(E)** HBV RNA. GAPDH acted as an internal control. We silenced OPN by specific siRNA transfection in soft (2 kPa) and stiff (25 kPa) gel-attached HBV-transfected HepG2.2.15 cells, and to mimic overexpression, we treated only soft (2 kPa) gel-attached HBV transfected HepG2.2.15 cells with rOPN for 24hrs. RT-PCR analysis was done for mRNA expression of USP18 **(F, G)**. GAPDH acted as an internal control for RT-PCR analysis. **(H)** USP18 protein was measured by immunoblotting and β-actin was also used as an internal control. Data are from three independent experiments presented as Mean ± SEM. Bars marked with the same letter are not significantly different from each other; bars with different letters are significantly different (P ≤0.05).

To prove that OPN downregulates ISGs, we treated soft (2 kPa) gel-attached HBV-transfected HepG2.2.15 cells with rOPN (to mimic overexpression). The rOPN treatment of soft gel-attached HepG2.2.15 cells successfully impaired the IFNα signaling by suppressing the mRNA expression of ISGs, *OAS1, APOBEC3G*, and *ISG15* as well as the protein expression of pSTAT-1 in response to IFNα, resulting in increased HBV RNA levels when compared to soft gel-attached cells not exposed to OPN (**Figures 3A-F**). This experiment (**Figure 3**) was performed on soft gels since OPN expression is higher in stiff gel vs soft gel. Thus, we tried to mimic OPN levels in the cells plated on stiff gels by overexpressing OPN (treatment with recombinant OPN) in cells plated on soft gels and then measured ISG expressions. In addition, we silenced OPN by specific siRNA transfection, (to mimic knock-down) in soft (2 kPa) and stiff (25 kPa) gel-attached HBV-transfected HepG2.2.15 cells. Silencing OPN (**Figure 4A**) enhanced anti-viral gene activation with reduced HBV RNA levels. IFNα significantly suppressed the HBV RNA levels (**Figures 4B-E**).

### Possible mechanisms of the regulation of HBV infection markers by OPN in fibrotic stiffness gel-attached HBV+ transfected HepG2.2.15 cells

To investigate how OPN regulates the levels of HBV infection markers in fibrotic stiffness (25 kPa) gel-attached HBV-transfected cells, we measured the *USP18* mRNA expression (a negative regulator of IFN signaling) in the presence of IFNα either in OPN-silenced soft (2 kPa) and stiff (25 kPa) gel-attached HepG2.2.15 cells or in rOPN-treated soft (2 kPa) gel-attached HepG2.2.15 cells. We observed that OPN silencing significantly (p<0.05) decreased *USP18* mRNA levels (**Figure 4F**) in fibrotic stiffness gel-attached HepG2.2.15 cells, whereas rOPN treatment did not increase the *USP18* mRNA expression in soft gel-attached HepG2.2.15 cells when compared to IFNα-treated group (**Figure 4G**). We performed this experiment (4G) on soft gels. Since we found that OPN expression was higher on stiff gel vs soft gel, we tried to mimic this by overexpressing OPN (treatment with recombinant OPN) in cells plated on the soft gel. In addition, we observed that recombinant OPN treatment significantly increased the USP18 protein expression in soft gel attached HepG2.2.15 cells when compared to control cells (**Figure 4H**).

### *In vivo* evidence of the role of liver tissue stiffness in HBV infection pathogenesis

**Figure 5:**
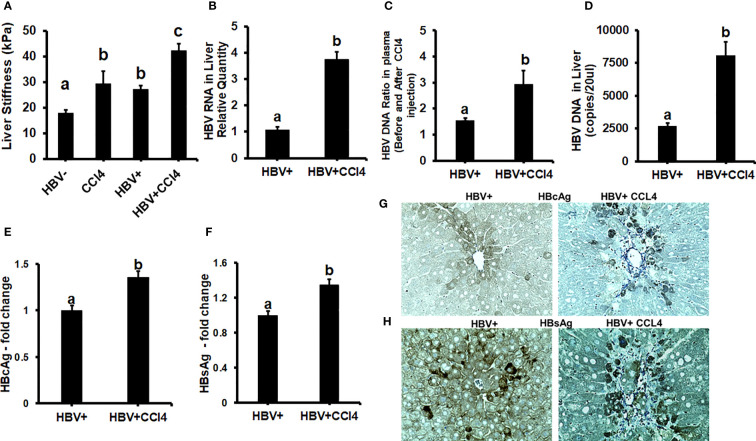
Increased liver tissue stiffness regulates HBV replication/enhanced expression of HBV markers in livers of CCl4-administered HBV+ transgenic mice (HBVTg): **(A)** Liver tissue stiffness was measured using the Biotester (CellScale Biomaterial Testing); **(B)** HBV RNA levels were measured by Real-Time PCR; **(C)** HBV DNA levels in serum by COBAS Amplicor HBV monitor test; **(D)** HBV DNA levels in the liver was measured by ddPCR; **(E-H)** Immunohistochemical detection of HBcAg, and HBsAg was visualized using a Keyence BZ-X810 fluorescence microscope. Staining intensity in the captured images was quantified using the Keyence BZ-X810 Analyzer software. At least, 10 nonoverlapping fields per low magnification (20X) section were analyzed. GAPDH was used as an internal control for HBV RNA and HBV DNA. Data are given as Mean ± SEM (n = 4). The data bars marked with the same alphabetical letter are considered not significantly different, while those marked with differing alphabetical letters are considered significantly different from each other (p ≤ 0.05).

**Figure 6:**
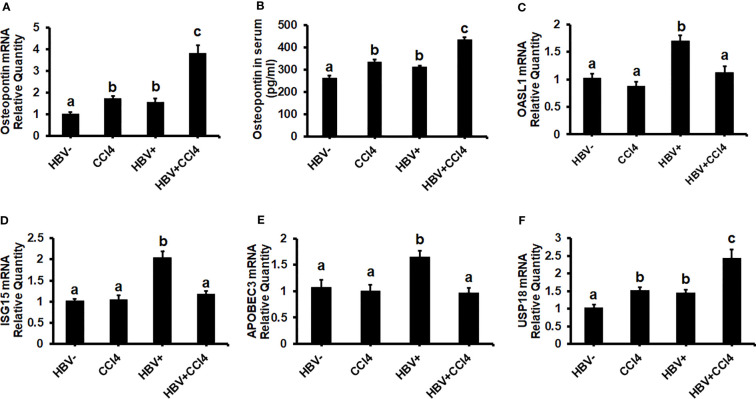
Increased liver tissue stiffness promotes osteopontin which suppresses Interferon-Stimulated Gene (ISG) expression in the livers of CCl4-administered HBVTg mice: Liver fibrosis was induced by injecting CCl4 for 6 weeks in C57Bl/6 parental and HBV+ transgenic (HBVTg) mice. RT-PCR analysis was done for mRNA expression of OPN, IFN-induced genes, and USP18. **(A)** Osteopontin (OPN); **(C)** 2’-5’ oligoadenylate synthetase-like 1 (OASL1); **(D)** Interferon-stimulated gene 15 (ISG15); **(E)** Apolipoprotein B Editing Complex (APOBEC3); **(F)** USP18. **(B)** OPN protein levels were measured by Elisa in serum. Data are expressed as Mean ± SEM (n =4). The data bars marked with the same alphabetical letter are considered not significantly different, while those marked with differing alphabetical letters are considered significantly different from each other (p ≤ 0.05).

To confirm our *in vitro* findings that an increased matrix stiffness regulates HBV-replication/enhanced expression of HBV markers, liver fibrosis was induced by injecting CCl4 for 6 weeks in C57Bl/6 parental and HBV+ transgenic (HBVTg) mice. Liver tissue stiffness was measured using the Biotester (CellScale Biomaterial Testing). We observed that the liver stiffness was higher (2-fold) in CCl4-administered HBVTg mice as well as in wild-type mice compared with control mice of each group (**Figure 5A**). As shown in **Figure 5A**, the ex vivo liver tissue stiffness of control mice reads 17 kPa. This is due to possible alterations in mechanical properties in ex vivo tissues due to perfusion pressure, tissue degradation, and boundary condition compared to *in vivo* environment. Also, this study indicates that the mechanical properties change drastically within only several minutes postmortem. (37) To account for this deviation, we processed all the conditions on the same day to measure the stiffness and the comparison in stiffness to indicate the changes in the original stiffness under each treatment condition. Based on the above-mentioned changes, the liver tissue stiffness in wild-type control mice is considered to represent the stiffness in the healthy liver, while the CCl4-induced increase in liver stiffness corresponds to fibrotic stiffness. Interestingly, control HBVTg mice showed higher liver tissue stiffness when compared to control wild-type mice. To determine whether the observed increased liver tissue stiffness plays a role in the up-regulation of HBV infection markers in CCl4-administered HBVTg mice, we measured HBV RNA and DNA in mouse livers and HBV DNA levels in serum by COBAS Amplicor HBV monitor test and performed immunohistochemical analysis of HBcAg and HBsAg in the liver (**Figures 5B-H**). These HBV-specific markers were significantly elevated (p ≤ 0.05) in the livers of CCl4-administered HBVTg mice. In addition, we found that both mRNA and protein expressions of OPN were significantly increased in the livers and serum of CCl4-administered control and HBVTg mice when compared to the respective control wild-type and control HBVTg mice (**Figures 6A, B**).

### Liver tissue stiffness suppresses ISGs expression in the livers of CCl4-administered HBVTg mice

Since in our *in vitro* study we found that increased stiffness induced up-regulation of OPN and regulates HBV-infection markers due to the suppression of IFNα-inducible anti-viral genes, we wanted to check whether an increase in liver tissue stiffness exerts the same effect in CCl4-administered HBVTg mice. To this end, we measured the mRNA expression of ISGs, OASL1, ISG15, and APOBEC. These mRNA expressions were significantly (P<0.05) decreased in the livers of CCl4-administered HBVTg mice when compared to control HBVTg mice (**Figures 6C-E**). We also found that the mRNA expression level of the negative regulator of IFNα signaling, *USP1*8, was increased both in the CCl4-administered wild type and HBVTg mice (**Figure 6F**). There was no alteration in the mRNA expression of ISGs in CCl4-administered wild-type mice when compared to control wild-type mice.

### CCl4 induces hepatic fibrosis in HBV- and HBV+ transgenic mice

**Figure 7:**
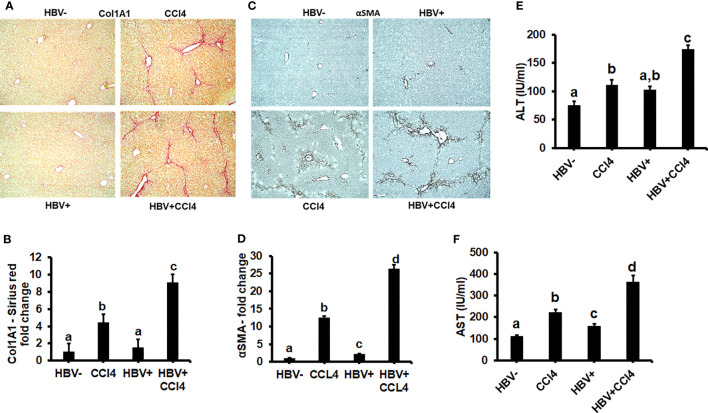
CCl4 induces hepatic fibrosis in HBV- and HBV+ transgenic mice: Liver fibrosis was induced by injecting CCl4 for 6 weeks in C57Bl/6 parental and HBV+ transgenic (HBVTg) mice. **(A, B)** Collagen accumulation was visualized using sections of paraffin-embedded liver tissue which were subjected to picrosirius red staining; **(C, D)** α-Smooth muscle actin (α-SMA) protein levels were detected by immunohistochemical detection. Pictures were captured using a Keyence BZ-X810 fluorescence microscope. Staining intensity in the captured images was quantified using the Keyence BZ-X810 Analyzer software. At least, 10 nonoverlapping fields per low magnification (20X) section were analyzed. **(E, F)** ALT and AST (serum enzymes) activities were determined in the serum of control and HBV-infected CCl4-administered mice. Data are expressed as Mean ± SEM (n =4). The data bars marked with the same alphabetical letter are considered not significantly different, while those marked with differing alphabetical letters are considered significantly different from each other (p ≤ 0.05).

**Figure 8:**
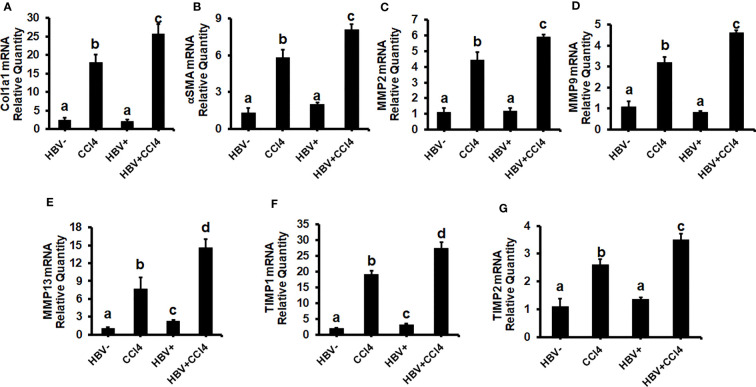
CCl4-administered HBV- and HBV+ transgenic mice show an increase in pro-fibrotic markers: Liver fibrosis was induced by injecting CCl4 for 6 weeks in C57Bl/6 parental and HBV+ transgenic (HBVTg) mice. RT-PCR analysis was done for mRNA expression of **(A)** Col1A1; **(B)** α-SMA; **(C)** MMP2; **(D)** MMP9; **(E)** MMP13; **(F)** TIMP1; and **(G)** TIMP2. Data are expressed as Mean ± SEM (n =4). The data bars marked with the same alphabetical letter are considered not significantly different, while those marked with differing alphabetical letters are considered significantly different from each other (p ≤ 0.05).

The livers were analyzed by picrosirius staining and immunohistochemical analysis to identify the collagen content and α-smooth muscle actin (SMA) (**Figures 7A-D**). The mice receiving CCl4 had a 2-fold increase in collagen and SMA expression when compared to control (unmanipulated) mice, which confirmed that fibrosis had been established. In addition, there was an increase in ALT and AST levels in CCl4-treated mice when compared to control mice (**Figures 7E, F**). HBVTg control mice showed elevated AST levels when compared to control wild-type mice. Total body and liver weights were not significantly different between the groups (Data not shown). The rate of collagen I gene (*Col1A1*) and *SMA* expression was determined using RT-PCR. A significant increase (p ≤ 0.05) in *Col1A1* and *SMA* expression was established by CCl4 treatment in wild-type and HBVTg mice (**Figures 8A, B**). Proteolysis is a major mechanism in the regulation of collagen levels. MMPs degrade collagen and other extracellular matrix proteins and are also regulated by their endogenous inhibitors and tissue inhibitors of matrix metalloproteinases (TIMPs). We found that expression of MMPs (MMP2, 9, and 13) and TIMPs (TIMP1 and 2) were significantly increased (p ≤ 0.05) in CCl4-exposed wild-type and HBVTg mice when compared to control mice of these types (**Figures 8C-G**).

## Discussion

Though adaptive immune response by HBV-specific CD8+ T cell (cytotoxic T-lymphocytes-CTLs) is largely responsible for clearing HBV-infected hepatocytes and disease pathogenesis (38), we cannot ignore the host’s first line of defense, the innate immune response. (39, 40) During HBV infection, antiviral innate immunity is activated when host-pathogen recognition receptors (PRRs) recognize the pathogen-associated molecular patterns (PAMPs) of the virus. (41, 42) The liver is an important immune organ, enriched with innate immune cells mounting a rapid and robust immune response. (43, 44) Hepatocytes are responsible for the biosynthesis of 80–90% of innate immune proteins. (45, 46) Furthermore, dysfunctional immune responses play an essential role in liver inflammation and persistent HBV infection. (4, 47–49) The increase in liver stiffness is driven by the inflammatory process, significantly pronounced CHB, (50), and correlates with liver decompensation, development of hepatocellular carcinoma, and patient survival. Thus, the measurement of liver stiffness is a non-invasive tool for monitoring CHB progression (51) to determine liver fibrosis and evaluate the efficiency of the treatment. (52) In this study, we aimed to investigate the role of increased liver stiffness in HBV infection pathogenesis addressed by both *in vitro* and *in vivo* experiments. Here, we hypothesized that matrix stiffness enhances OPN levels to limit anti-viral ISG activation and promote the increase in HBV infectivity, thereby contributing to end-stage liver disease progression.

For *in vitro* studies, an innovative biomimetic platform called BEASTS (Bio-Engineered Adhesive Siloxane substrate with Tunable Stiffness) was developed by combining a polydimethylsiloxane (PDMS) substrate with polyelectrolyte multilayer film-coating technology. This enabled us to engineer substrates that mimic both physiologic (2 kPa) and fibrotic liver stiffness (25 kPa) on which we cultured HepG2.2.15 cells to study their behavior under these two conditions. We found that with increasing matrix stiffness, there was upregulation of HBV markers and OPN levels. This might be due to the ability of OPN to suppress innate immunity that controls viral replication in HBV-transfected HepG2.2.15 cells. One of the most potent mechanisms of anti-viral defense is the induction of ISGs in hepatocytes activated by IFNα via the JAK-STAT1-2 pathways. Therefore, we tested IFNα signaling via the JAK-STAT1 pathway in cells plated on healthy and fibrotic stiffness gels. We found that both upstream STAT1 phosphorylation and downstream activation of ISGs, namely, OAS1, ISG15, and APOBEC3G, were impaired in fibrotic stiffness gel-attached HBV-transfected HepG2.2.15 cells. In addition, we observed that USP18, a negative regulator of IFN signaling, was significantly increased at both the mRNA and protein levels in fibrotic stiffness gel-attached HBV-transfected cells. Importantly, overexpression of OPN (exposure of cells to rOPN) suppressed ISGs and increased HBV RNA levels. In addition, OPN silencing by specific siRNA transfection increased ISG expression and decreased the HBV RNA levels in fibrotic stiffness gel-attached, IFNα-treated, HBV-transfected cells, suggesting that OPN plays a partial role in impairing innate immune response via ISGs suppression. Thus, when cells are under fibrotic stiffness conditions (attached to stiff gels), this leads to an increase in HBV RNA levels.

Our findings were supported by other studies showing that higher matrix stiffness upregulates OPN expression in HCC cells. (19) In addition, OPN concentrations were increased in CHB patients (53), and OPN was identified as a biomarker of liver fibrosis in hepatitis B. (54) The suppression of STAT1 phosphorylation by increased OPN found in our study is not unique to only chronic HBV infection. In fact, similar to our findings in HBV-infected cells, for HCV infection, a study has reported that OPN-overexpressing Huh7 cells significantly increased HCV replication by suppressing IFNα stimulated genes (ISGs) via the inhibition of STAT-1 phosphorylation and degradation. (55) Moreover, the modulation of OPN levels by overexpressing or silencing OPN increased HCV and HIV replication in Huh 7.5 cells or primary macrophages, respectively. (56, 57) Importantly, a study has reported that OPN drives HBV replication and HBV-driven fibrogenesis. (58) However, these studies provided no link between enhanced liver stiffness and upregulation of HCV-HIV markers. Interestingly, our previous studies demonstrated an increased HCV-HIV mRNA expression and apoptosis induction in hepatocytes plated on fibrotic stiffness. (26) While hepatocyte apoptosis has been shown to induce pro-fibrotic activation of HSC in these infections (59–61), HBV infection seems to have different properties since we observed no increased apoptosis in cells transfected with HBV. At that point, we were not able to identify the mechanisms of increased viral infectivity in hepatocytes under fibrotic conditions and to link an increased liver stiffness to OPN upregulation and suppression of innate immunity. However, in the current study, we observed a decrease in STAT-1 phosphorylation in stiff gel-attached HBV-transfected cells, and OPN overexpression suppressed ISGs, thereby increasing HBV RNA levels. As a next step, we investigated the relationship between OPN levels and USP18. We measured the *USP18* mRNA levels in rOPN-treated and OPN-silenced stiff gel-attached HBV-transfected cells (under fibrotic conditions). We found that only OPN-silenced cells show a decrease in *USP18* mRNA, while rOPN did not further increase the USP18 mRNA levels. Interestingly, rOPN treatment significantly increased the USP18 protein levels. This finding indicates that OPN partially contributes to the elevation of USP18 and suggests that while OPN affects USP18 expression, this protein is not a direct target for OPN and requires some intermediate “inserts”. Our *in vitro* study provides evidence that fibrotic stiffness induced OPN expression in hepatocytes, causing upregulation of HBV replication and HBV markers due to decreased innate immunity. In fact, it has been reported that in HBV infection, chronic liver inflammation co-exists with impaired antiviral immunity though the exact mechanism has not been previously discovered (62).

As proof of concept that liver stiffness-induced OPN releases increased HBV replication, we conducted an *in vivo* experiment using C57Bl/6 parental (wild type) and HBV+ transgenic (HBVTg) mice. Although recent studies have shown the role of tissue stiffness in liver fibrogenesis, little is known about its role in HBV infection pathogenesis. This is the first *in vivo* study where we investigated the role of liver stiffness in HBV infection pathogenesis using HBVTg mice. To mimic fibrotic liver, hepatic fibrosis was established by administering CCl4 for 6 weeks. Here, we observed an enhanced liver stiffness and an upregulation of HBV-specific markers with an increase in OPN expression in livers and serum of CCl4-administered HBVTg mice. Interestingly, the expression of IFN type 1-inducible anti-viral genes was decreased with an upregulation in *USP18* mRNA expression in fibrotic HBVTg mice. ALT and AST levels and fibrosis markers were higher in CCl4-administered HBVTg mice. The findings from this *in vivo* study were supported by our *in vitro* results as well as clinical studies by others, demonstrating that a high serum OPN level and high liver stiffness in chronic HBV infection predict the failure of virological response and hepatic fibrosis regression. (63) It has been reported that hepatic stiffness was important for monitoring the outcomes of hepatic fibrosis in HBV antiviral therapy, and long-term viral inhibition was correlated with the outcomes of hepatic fibrosis. (64) The present study provides valuable evidence on the importance of liver stiffness, OPN, and their relationship in the regulation of innate immunity in advanced HBV infection.

We cannot exclude that in the presence of macrophages and hepatic stellate cells, OPN release will be more potent and co-culture of hepatocytes with these cells would provide a more robust effect on hepatocyte infectivity. In fact, we observed it in our *in vivo* studies, where the magnitude of OPN response in the liver was higher than in hepatocyte monoculture. However, here, we tried to find an explanation for the up-regulation of HBV markers in infected hepatocytes plated on the stiff gel, and we believe that it is related to OPN-induced suppression of anti-viral ISGs. This has been demonstrated by overexpressing and knocking down OPN in HBV+ HepG2.2.15 cells. In future studies, we are planning to perform co-culture experiments for liver parenchymal and non-parenchymal cells or to look at their cell-to-cell interactions using liver organoids.

**Figure 9:**
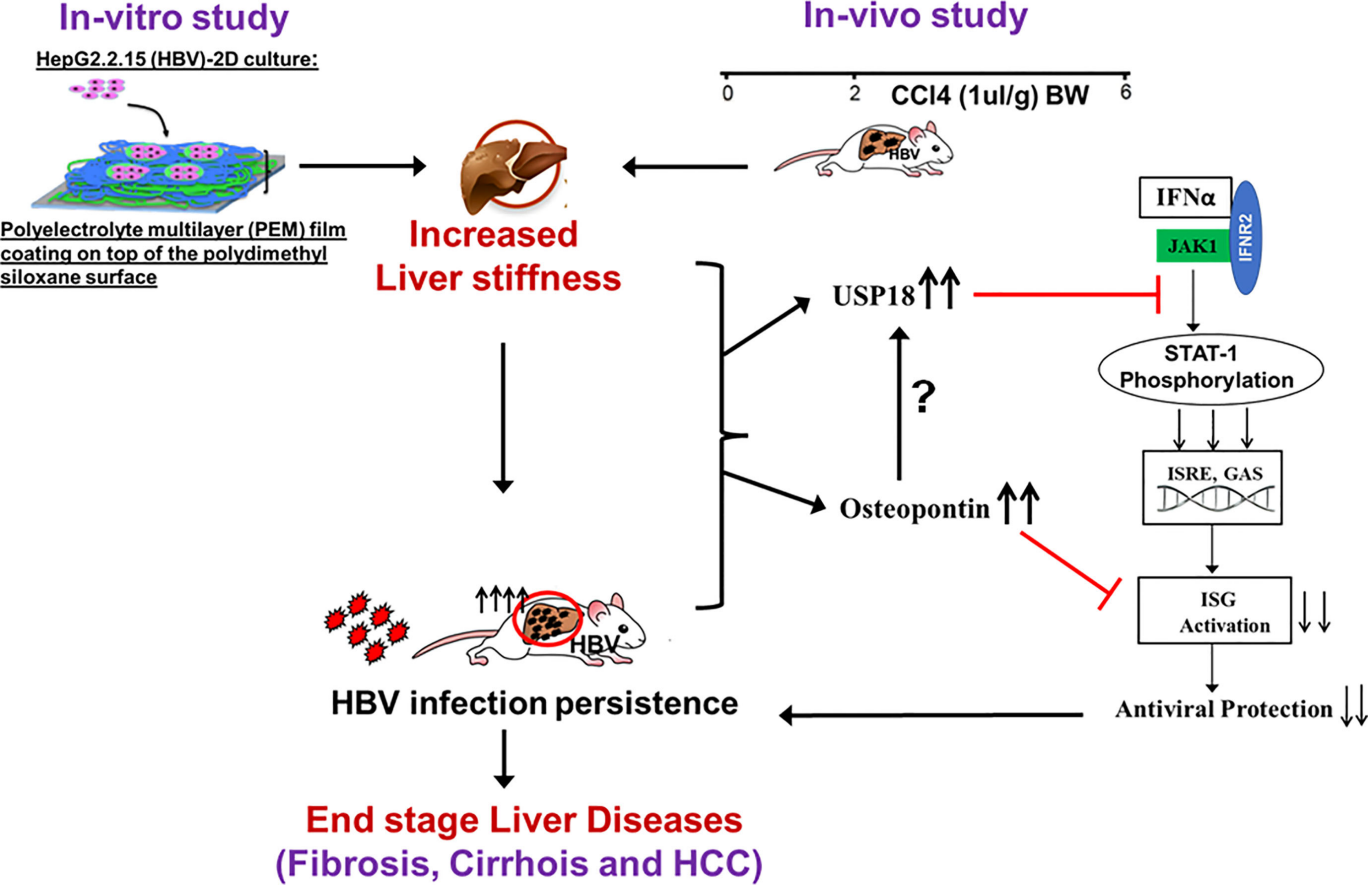
The proposed model for increased liver stiffness inducing hepatitis B progression and end-stage liver diseases: *In vitro* and *In vivo* data show that an increased matrix stiffness upregulates the release of OPN, which limits the IFNα-induced innate immune response by suppressing the STAT-1 and ISGs activation, thereby upregulating the expression of HBV markers and inflammation and promoting the development of end-stage liver diseases. This provides an optional explanation for continued and fast HBV infection progression when the disease comes to the liver fibrosis stage.

Based on our *in vitro* and *in vivo* data, we conclude that an increased matrix stiffness upregulates the release of OPN, which limits the IFNα- induced innate immune response by suppressing the STAT-1 and ISGs activation, thereby upregulating the expression of HBV markers and promoting the development of end-stage liver diseases (**Figure 9**). This provides an optional explanation for continued and fast HBV infection progression when the disease comes to the liver fibrosis stage.

## Data availability statement

The original contributions presented in the study are included in the article/supplementary material. Further inquiries can be directed to the corresponding author.

## Ethics statement

The animal study was reviewed and approved by Institutional Animal Care and Use Committee of University of Nebraska Medical Center.

## Author contributions

MG, GB, YM, and WW: conducted experiments and evaluated data; SK: provided materials for the experiment and carried out the liver tissue stiffness measurement; MG and SK: interpreted results and information obtained from experiments; KK, LP, and NO: edited the manuscript, provided suggestions to improve the project, and performed corrections; MG: obtained grant funding, developed and designed research project goals and experiments, and performed the final edit of the manuscript. All authors contributed to the article and approved the submitted version.

## References

[1] El-Serag HB. Epidemiology of viral hepatitis and hepatocellular carcinoma. Gastroenterology (2012) 142:1264–1273 e1261. doi: 10.1053/j.gastro.2011.12.061 22537432 PMC3338949

[2] Locarnini S Hatzakis A Chen DS Lok A. Strategies to control hepatitis B: Public policy, epidemiology, vaccine and drugs. J Hepatol (2015) 62:S76–86. doi: 10.1016/j.jhep.2015.01.018 25920093

[3] Lim CJ Lee YH Pan L Lai L Chua C Wasser M. Multidimensional analyses reveal distinct immune microenvironment in hepatitis B virus-related hepatocellular carcinoma. Gut (2019) 68:916–27. doi: 10.1136/gutjnl-2018-316510 29970455

[4] Revill PA Chisari FV Block JM Dandri M Gehring AJ Guo H. A global scientific strategy to cure hepatitis B. Lancet Gastroenterol Hepatol (2019) 4:545–58. doi: 10.1016/S2468-1253(19)30119-0 PMC6732795 30981686

[5] Iannacone M Guidotti LG. Immunobiology and pathogenesis of hepatitis B virus infection. Nat Rev Immunol (2022) 22:19–32. doi: 10.1038/s41577-021-00549-4 34002067

[6] Childs L Roesel S Tohme RA. Status and progress of hepatitis B control through vaccination in the South-East Asia Region 1992-2015. Vaccine (2018) 36:6–14. doi: 10.1016/j.vaccine.2017.11.027 29174317 PMC5774012

[7] Shin EC Sung PS Park SH. Immune responses and immunopathology in acute and chronic viral hepatitis. Nat Rev Immunol (2016) 16:509–23. doi: 10.1038/nri.2016.69 27374637

[8] Tang CM Yau TO Yu J. Management of chronic hepatitis B infection: current treatment guidelines, challenges, and new developments. World J Gastroenterol (2014) 20:6262–78. doi: 10.3748/wjg.v20.i20.6262 PMC4033464 24876747

[9] Inoue T Tanaka Y. Novel biomarkers for the management of chronic hepatitis B. Clin Mol Hepatol (2020) 26:261–79. doi: 10.3350/cmh.2020.0032 PMC7364351 32536045

[10] Levrero M Zucman-Rossi J. Mechanisms of HBV-induced hepatocellular carcinoma. J Hepatol (2016) 64:S84–S101. doi: 10.1016/j.jhep.2016.02.021 27084040

[11] Mcmahon BJ. The natural history of chronic hepatitis B virus infection. Hepatology (2009) 49:S45–55. doi: 10.1002/hep.22898 19399792

[12] Parikh P Ryan JD Tsochatzis EA. Fibrosis assessment in patients with chronic hepatitis B virus (HBV) infection. Ann Transl Med (2017) 5:40. doi: 10.21037/atm.2017.01.28 28251119 PMC5326648

[13] Georges PC Hui JJ Gombos Z Mccormick ME Wang AY Uemura M. Increased stiffness of the rat liver precedes matrix deposition: implications for fibrosis. Am J Physiol Gastrointest Liver Physiol (2007) 293:G1147–1154. doi: 10.1152/ajpgi.00032.2007 17932231

[14] Masuzaki R Tateishi R Yoshida H Yoshida H Sato S Kato N. Risk assessment of hepatocellular carcinoma in chronic hepatitis C patients by transient elastography. J Clin Gastroenterol (2008) 42:839–43. doi: 10.1097/MCG.0b013e318050074f 18668703

[15] Liu L Nie Y Zhang Y Liu Q Zhu X. Liver Stiffness Is a Predictor of Rebleeding in Patients With Hepatitis B-Related Cirrhosis: A Real-World Cohort Study. Front Med (Lausanne) (2021) 8:690825. doi: 10.3389/fmed.2021.690825 34395474 PMC8355367

[16] Tschumperlin DJ Ligresti G Hilscher MB Shah VH. Mechanosensing and fibrosis. J Clin Invest (2018) 128:74–84. doi: 10.1172/JCI93561 29293092 PMC5749510

[17] Lampi MC Reinhart-King CA. Targeting extracellular matrix stiffness to attenuate disease: From molecular mechanisms to clinical trials. Sci Transl Med (2018) 10:eaao0475. doi: 10.1126/scitranslmed.aao0475 29298864

[18] Honsawek S Chayanupatkul M Chongsrisawat V Vejchapipat P Poovorawan Y. Increased osteopontin and liver stiffness measurement by transient elastography in biliary atresia. World J Gastroenterol (2010) 16:5467–73. doi: 10.3748/wjg.v16.i43.5467 PMC2988241 21086566

[19] You Y Zheng Q Dong Y Wang Y Zhang L Xue T. Higher Matrix Stiffness Upregulates Osteopontin Expression in Hepatocellular Carcinoma Cells Mediated by Integrin beta1/GSK3beta/beta-Catenin Signaling Pathway. PloS One (2015) 10:e0134243. doi: 10.1371/journal.pone.0134243 26280346 PMC4539226

[20] Wen Y Jeong S Xia Q Kong X. Role of Osteopontin in Liver Diseases. Int J Biol Sci (2016) 12:1121–8. doi: 10.7150/ijbs.16445 PMC4997056 27570486

[21] Liu HB Chen QY Wang XY Zhang LJ Hu LP Harrison TJ. Infection with Hepatitis B Virus May Increase the Serum Concentrations of Osteopontin. Intervirology (2021) 64:126–34. doi: 10.1159/000513687 PMC8491474 33735879

[22] Ramaiah SK Rittling S. Pathophysiological role of osteopontin in hepatic inflammation, toxicity, and cancer. Toxicol Sci (2008) 103:4–13. doi: 10.1093/toxsci/kfm246 17890765

[23] Syn WK Choi SS Liaskou E Karaca GF Agboola KM Oo YH. Osteopontin is induced by hedgehog pathway activation and promotes fibrosis progression in nonalcoholic steatohepatitis. Hepatology (2011) 53:106–15. doi: 10.1002/hep.23998 PMC3025083 20967826

[24] Morales-Ibanez O Dominguez M Ki SH Marcos M Chaves JF Nguyen-Khac E. Human and experimental evidence supporting a role for osteopontin in alcoholic hepatitis. Hepatology (2013) 58:1742–56. doi: 10.1002/hep.26521 PMC3877722 23729174

[25] Natarajan V Berglund EJ Chen DX Kidambi S. Substrate stiffness regulates primary hepatocyte functions. RSC Adv (2015) 5:80956–66. doi: 10.1039/C5RA15208A PMC7392243 32733675

[26] Ganesan M Dagur RS Makarov E Poluektova LI Kidambi S Osna NA. Matrix stiffness regulate apoptotic cell death in HIV-HCV co-infected hepatocytes: Importance for liver fibrosis progression. Biochem Biophys Res Commun (2018) 500:717–22. doi: 10.1016/j.bbrc.2018.04.142 PMC6863049 29679566

[27] Kidambi S. Stiffness and Hepatocytes Function *In Vitro* . In book: Liver Elastography (2020), 645–60. doi: 10.1007/978-3-030-40542-7_55

[28] Natarajan V Moeller M Casey CC Harris EN Kidambi S. Matrix Stiffness Regulates Liver Sinusoidal Endothelial Cell Function Mimicking Responses in Fatty Liver Disease. bioRxiv. (2020). doi: 10.1101/2020.01.27.921353

[29] Natarajan V Moeun Y Kidambi S. Exploring Interactions between Primary Hepatocytes and Non-Parenchymal Cells on Physiological and Pathological Liver Stiffness. Biol (Basel) (2021) 10:408. doi: 10.3390/biology10050408 PMC8147966 34063016

[30] Zhao R Wang TZ Kong D Zhang L Meng HX Jiang Y. Hepatoma cell line HepG2.2.15 demonstrates distinct biological features compared with parental HepG2. World J Gastroenterol (2011) 17:1152–9. doi: 10.3748/wjg.v17.i9.1152 PMC3063907 21448419

[31] Ganesan M Krutik VM Makarov E Mathews S Kharbanda KK Poluektova LY. Acetaldehyde suppresses the display of HBV-MHC class I complexes on HBV-expressing hepatocytes. Am J Physiol Gastrointest Liver Physiol (2019) 317:G127–40. doi: 10.1152/ajpgi.00064.2019 PMC6734374 31141391

[32] Ganesan M Mathews S Makarov E Petrosyan A Kharbanda KK Kidambi S. Acetaldehyde suppresses HBV-MHC class I complex presentation on hepatocytes via induction of ER stress and Golgi fragmentation. Am J Physiol Gastrointest Liver Physiol (2020) 319:G432–42. doi: 10.1152/ajpgi.00109.2020 PMC7654643 32755306

[33] Ganesan M Wang W Mathews S Makarov E New-Aaron M Dagur RS. Ethanol attenuates presentation of cytotoxic T-lymphocyte epitopes on hepatocytes of HBV-infected humanized mice. Alcohol Clin Exp Res (2022) 46:40–51. doi: 10.1111/acer.14740 34773268 PMC8799491

[34] Constandinou Cc HN Iredale J. Modeling liver fibrosis in rodents. In: Varga J Brenner DA Phan SH, editors. Fibrosis Research: Methods and Protocols. Totowa, NJ: Humana Press (2005). p. 237–50. 10.1385/1-59259-940-0:237 16118456

[35] Bennett RG Heimann DG Singh S Simpson RL Tuma DJ. Relaxin decreases the severity of established hepatic fibrosis in mice. Liver Int (2014) 34:416–26. doi: 10.1111/liv.12247 PMC3843971 23870027

[36] Junqueira LC Bignolas G Brentani RR. Picrosirius staining plus polarization microscopy, a specific method for collagen detection in tissue sections. Histochem J (1979) 11:447–55. doi: 10.1007/BF01002772 91593

[37] Weickenmeier J Kurt M Ozkaya E De Rooij R Ovaert TC Ehman RL. Brain stiffens post mortem. J Mech Behav BioMed Mater (2018) 84:88–98. doi: 10.1016/j.jmbbm.2018.04.009 29754046 PMC6751406

[38] Gehring AJ Protzer U. Targeting Innate and Adaptive Immune Responses to Cure Chronic HBV Infection. Gastroenterology (2019) 156:325–37. doi: 10.1053/j.gastro.2018.10.032 30367834

[39] Megahed F Zhou X Sun P. The Interactions between HBV and the Innate Immunity of Hepatocytes. Viruses (2020) 12:285. doi: 10.3390/v12030285 32151000 PMC7150781

[40] Xu C Chen J Chen X. Host Innate Immunity Against Hepatitis Viruses and Viral Immune Evasion. Front Microbiol (2021) 12:740464. doi: 10.3389/fmicb.2021.740464 34803956 PMC8598044

[41] Lester SN Li K. Toll-like receptors in antiviral innate immunity. J Mol Biol (2014) 426:1246–64. doi: 10.1016/j.jmb.2013.11.024 PMC3943763 24316048

[42] Streicher F Jouvenet N. Stimulation of Innate Immunity by Host and Viral RNAs. Trends Immunol (2019) 40:1134–48. doi: 10.1016/j.it.2019.10.009 31735513

[43] Gao B. Basic liver immunology. Cell Mol Immunol (2016) 13:265–6. doi: 10.1038/cmi.2016.09 PMC4856812 27041634

[44] Ringelhan M Pfister D O'connor T Pikarsky E Heikenwalder M. The immunology of hepatocellular carcinoma. Nat Immunol (2018) 19:222–32. doi: 10.1038/s41590-018-0044-z 29379119

[45] Gao B Jeong WI Tian Z. Liver: An organ with predominant innate immunity. Hepatology (2008) 47:729–36. doi: 10.1002/hep.22034 18167066

[46] Gao B Seki E Brenner DA Friedman S Cohen JI Nagy L. Innate immunity in alcoholic liver disease. Am J Physiol Gastrointest Liver Physiol (2011) 300:G516–525. doi: 10.1152/ajpgi.00537.2010 PMC3774265 21252049

[47] Rehermann B. Pathogenesis of chronic viral hepatitis: differential roles of T cells and NK cells. Nat Med (2013) 19:859–68. doi: 10.1038/nm.3251 PMC4482132 23836236

[48] Peeridogaheh H Meshkat Z Habibzadeh S Arzanlou M Shahi JM Rostami S. Current concepts on immunopathogenesis of hepatitis B virus infection. Virus Res (2018) 245:29–43. doi: 10.1016/j.virusres.2017.12.007 29273341

[49] Rehermann B Thimme R. Insights From Antiviral Therapy Into Immune Responses to Hepatitis B and C Virus Infection. Gastroenterology (2019) 156:369–83. doi: 10.1053/j.gastro.2018.08.061 PMC6340757 30267712

[50] Branchi F Conti CB Baccarin A Lampertico P Conte D Fraquelli M. Non-invasive assessment of liver fibrosis in chronic hepatitis B. World J Gastroenterol (2014) 20:14568–80. doi: 10.3748/wjg.v20.i40.14568 PMC4209524 25356021

[51] Oliveri F Coco B Ciccorossi P Colombatto P Romagnoli V Cherubini B. Liver stiffness in the hepatitis B virus carrier: a non-invasive marker of liver disease influenced by the pattern of transaminases. World J Gastroenterol (2008) 14:6154–62. doi: 10.3748/wjg.14.6154 PMC2761576 18985805

[52] Kavak S Kaya S Senol A Sogutcu N. Evaluation of liver fibrosis in chronic hepatitis B patients with 2D shear wave elastography with propagation map guidance: a single-centre study. BMC Med Imaging (2022) 22:50. doi: 10.1186/s12880-022-00777-7 35303822 PMC8932279

[53] Diao H Liu X Wu Z Kang L Cui G Morimoto J. Osteopontin regulates interleukin-17 production in hepatitis. Cytokine (2012) 60:129–37. doi: 10.1016/j.cyto.2012.06.287 22818182

[54] Zhao L Li T Wang Y Pan Y Ning H Hui X. Elevated plasma osteopontin level is predictive of cirrhosis in patients with hepatitis B infection. Int J Clin Pract (2008) 62:1056–62. doi: 10.1111/j.1742-1241.2007.01368.x 17537188

[55] Shirasaki T Honda M Yamashita T Nio K Shimakami T Shimizu R. The osteopontin-CD44 axis in hepatic cancer stem cells regulates IFN signaling and HCV replication. Sci Rep (2018) 8:13143. doi: 10.1038/s41598-018-31421-6 30177680 PMC6120883

[56] Brown A Islam T Adams R Nerle S Kamara M Eger C. Osteopontin enhances HIV replication and is increased in the brain and cerebrospinal fluid of HIV-infected individuals. J Neurovirol (2011) 17:382–92. doi: 10.1007/s13365-011-0035-4 PMC3331788 21556958

[57] Iqbal J Sarkar-Dutta M Mcrae S Ramachandran A Kumar B Waris G. Osteopontin Regulates Hepatitis C Virus (HCV) Replication and Assembly by Interacting with HCV Proteins and Lipid Droplets and by Binding to Receptors alphaVbeta3 and CD44. J Virol (2018) 92:e02116–17. doi: 10.1128/JVI.02116-17 29669827 PMC6002707

[58] Phillips S Mistry S Coombes J Hadzhiolova T Doornebal E Harris N. Osteopontin drives HBV replication and HBV-driven fibrogenesis and represents a novel therapeutic target to achieve functional cure in chronic hepatitis B. J Hepatol (2020) 73:S845. doi: 10.1016/S0168-8278(20)32133-4

[59] Ganesan M Poluektova LY Enweluzo C Kharbanda KK Osna NA. Hepatitis C Virus-Infected Apoptotic Hepatocytes Program Macrophages and Hepatic Stellate Cells for Liver Inflammation and Fibrosis Development: Role of Ethanol as a Second Hit. Biomolecules (2018) 8:113. doi: 10.3390/biom8040113 30322122 PMC6316463

[60] Ganesan M New-Aaron M Dagur RS Makarov E Wang W Kharbanda KK. Alcohol Metabolism Potentiates HIV-Induced Hepatotoxicity: Contribution to End-Stage Liver Disease. Biomolecules (2019) 9:851. doi: 10.3390/biom9120851 31835520 PMC6995634

[61] New-Aaron M Dagur RS Koganti SS Ganesan M Wang W Makarov E. Alcohol and HIV-Derived Hepatocyte Apoptotic Bodies Induce Hepatic Stellate Cell Activation. Biol (Basel) (2022) 11:1059. doi: 10.3390/biology11071059 PMC9312505 36101437

[62] Song H Tan G Yang Y Cui A Li H Li T. Hepatitis B Virus-Induced Imbalance of Inflammatory and Antiviral Signaling by Differential Phosphorylation of STAT1 in Human Monocytes. J Immunol (2019) 202:2266–75. doi: 10.4049/jimmunol.1800848 30842274

[63] Osman H Ghweil A Khodeary A. Role of Plasma Osteopontin Level as a Predictor of Hepatic Fibrosis Regression and Response to Antiviral Treatment in Patients with Chronic HBV or Chronic HCV Infection. Open J Gastroenterol (2018) 8:434–47. doi: 10.4236/ojgas.2018.812045

[64] Wu SD Liu LL Cheng JL Liu Y Cheng LS Wang SQ. Longitudinal monitoring of liver fibrosis status by transient elastography in chronic hepatitis B patients during long-term entecavir treatment. Clin Exp Med (2018) 18:433–43. doi: 10.1007/s10238-018-0501-x 29696462

